# P01-04 The importance of local organizational and leadership capacity to support Danish school heads in the implementation of a national physical activity school requirement

**DOI:** 10.1093/eurpub/ckac095.004

**Published:** 2022-08-29

**Authors:** Jonas Vestergaard Nielsen, Sofie Koch, Thomas Skovgaard

**Affiliations:** Department of Sports Science and Clinical Biomechanics, University of Southern Denmark, Odense M, Denmark

**Keywords:** Implementation, School setting, Qualitative methods, Leadership, Organizational capacity

## Abstract

**Background:**

Regular physical activity (PA) strengthens both the physical, psychological and social health in children and young people. Furthermore, research show that PA is beneficial for academic related outcomes. In 2014, the Danish government introduced a wide-ranging reform of primary and lower secondary education that applied to all public schools. A distinctive feature was that it became mandatory for schools to deliver an average of 45 minutes of daily PA. Local school heads and the school's capacity for change is considered key to deliver such a policy-driven requirement. Thus, the aim of this study is to explore the ability of schools to implement the stated requirement of 45 minutes of daily PA. There is special focus on the role and impact of leadership by school heads.

**Methods:**

Eleven semi-structured interviews were conducted across eleven Danish schools. Respondents were school staff with management responsibilities (leading teachers with managerial duties, deputy heads and school heads). Thematic analyses were performed, focusing on factors relating to local organizational and leadership capacity.

**Results:**

Three main factors were found to support the local leader's ability to the implement the mandatory daily PA: i) local school culture, values, and norms; ii) staff skillset and school resources; iii) existing work routines and systems.

**Conclusion:**

Results indicate that local school heads are central in converting the Danish school requirement of 45 minutes of daily PA into local action. Future PA programs could benefit from focusing specifically on engaging school heads, as they can both help advance broad ambitions into concrete goals, secure supportive structures and organize the implementation strategy within the local setting. In connection with this heads must be able to rely on sufficient organizational ad administrative components to ensure quality delivery. This entails building sufficient competencies among relevant staff groups on, for instance, how to incorporate PA in daily practice as well as allocate work hours for further development of such practices. Also, assigning a local PA ambassadors is highlighted as an important implementation factor. The ambassadors can help build and disseminate knowledge and support the school head's strategy and prioritization in relation to school-based PA.

